# Nature of antimicrobial resistance of pathogens causing urinary tract infection in Bangladesh: age and gender profiles

**DOI:** 10.1128/spectrum.02287-24

**Published:** 2025-04-25

**Authors:** Ferdous Jahan, Mohammed Anwer

**Affiliations:** 1 Department of Nephrology, Bangabandhu Sheikh Mujib Medical University 74464, Dhaka, Bangladesh; 2 Department of Physical Sciences, Independent University, Bangladesh (IUB), Dhaka, Bangladesh; Institut National de Santé Publique du Québec, Sainte-Anne-de-Bellevue, Québec, Canada

**Keywords:** urinary tract infection, antimicrobial resistance, antibiotics, multidrug resistance, Bangladesh

## Abstract

Over the years, the clinical management of urinary tract infections (UTIs) is continuously evolving as a challenge because of resistance to antibiotics. In addition to investigating the nature of antimicrobial resistance to antibiotics, this study will investigate the age and gender nature of patients having these resistances. Patients who were prescribed urine culture sensitivity tests as part of their treatment at a tertiary-level hospital in Dhaka, Bangladesh, were included as a sample. Urine culture was done following the guidelines of CLSI, and a sensitivity test was done following the Kirby-Bauer sensitivity method. About 55% of both males and females have been found to be infected with *Escherichia coli*, and about 15% were infected with *Klebsiella*. Other pathogens detected are *Enterococcus* and *Candida*. Age and gender nature of *E. coli* indicates that females are more infected below the age of 60 years, while males are more infected above this age. *E. coli* has exhibited the least resistance to Azithromycin, Netilmicin, and Amikacin. The resistance to Ciprofloxacin shows that females are more resistant until the age of 60 years, and males after that. Multidrug resistance shows an alarming result that about 20% of infected patients are resistant to nine antibiotics. This study clearly indicates that UTI is a continuously evolving problem, and its investigation should remain a continuous process.

**IMPORTANCE:**

Because of economic and social practices in Bangladesh, urinary tract infection is a continually evolving disease. The treatment requires relatively updated information about the behavior of the pathogens causing this infection. There is not sufficient information in Bangladesh about the age and gender nature of the infected patients, the infected pathogens, and their antibiotic resistance. Moreover, because of indiscriminate and uncontrolled use of antibiotics within the country, the nature of multidrug resistance of these pathogens is also important for the effective management of these patients. This research attempts to address these issues.

## INTRODUCTION

The difficulty of describing urinary tract infection (UTI) in an all-inclusive definition has been discussed recently in a review article (1). In general, UTI is considered as an infection that can occur in any part of the urinary system. For the purpose of UTI, the urinary system includes the urinary tract, urinary bladder, urethra, or kidneys (2). Globally, it is one of the common and leading causes of infection resulting in morbidities and mortalities (3–5). Conservative approximation estimates that around 150 million people around the world suffer from this infection causing substantial financial burden (6). When it comes to UTIs, females are the more vulnerable population, accounting for 60% of the global infected population (7, 8). Females are more susceptible because of the physiological and anatomical characteristics of their urethra (9).

Several previous studies have revealed that *Escherichia coli* is responsible for 80%–85% of all UTIs globally (3, 6, 7, 10). Other pathogens that have been found responsible for the infection include *Klebsiella, Proteus, Staphylococcus*, or *Enterococcus*. The infection mechanisms involve both community-acquired as well as hospital-acquired infections (4, 11).

In addition to having physiological, social, and financial impact, UTI has also been associated with psychological impact in terms of depression and anxiety (12, 13). Consequently, early diagnosis and treatment of the infection are necessary. However, in recent years, the emerging global trend of resistance of antibiotics to pathogens has become a challenge (14–16) in this regard. Indiscriminate use of antibiotics directly by individuals as well as secondarily through agricultural products—vegetables, meat, poultry, and fisheries—is contributing to the rise in this resistance. In recent years, this has become particularly alarming in the eastern part of India, Nepal, and Bangladesh (17, 18). In Bangladesh, in addition to all these factors, the availability of antibiotics without any doctor’s prescription is adding to the indiscriminate use of antibiotics—hence having a major contribution to antibiotic resistance in the country (19). Notwithstanding, this is having a major impact on the antimicrobial resistance of pathogens responsible for urinary tract infections as well.

Detailed knowledge regarding the nature of antimicrobial resistance of uropathogens in Bangladesh is very limited. In a recent study, previously published results from three studies over a span of 10 years were compared to show that there was a substantial change in antimicrobial resistance over this period (20). A similar study in China (15) over a span of 12 years also suggests a similar behavior. These studies suggest that studies looking into the antimicrobial resistance to uropathogens should preferably be conducted periodically over the years. This study was conducted keeping this target in mind.

The primary objective of this study is to investigate the nature of antimicrobial resistance to uropathogens to obtain an estimate of the present state of this problem. Past studies have suggested more details about the gender and age of the patients (15). Therefore, this study will report more detailed information on the antimicrobial resistance of uropathogens based on the gender and age of patients.

## MATERIALS AND METHODS

This is a retrospective (*a posteriori*) study conducted at a tertiary-level hospital in Dhaka, Bangladesh. To minimize sampling bias, patients were not specifically selected for the study. Instead, among the patients seeking treatment, those who presented with symptoms such as high fever with a burning sensation during urination, frequent or intense urges to urinate, or pain or pressure in the lower abdomen or back were suspected of having a urinary tract infection. These patients were advised to undergo a urinary culture sensitivity test. Only those who returned with their test results were included in the study. Female patients who were menstruating and those already on antibiotics were excluded from the study.

Standard urine sample collection procedure was utilized for acquiring the samples. Once collected, following standard operating procedure, the samples were transferred to the laboratory for testing within 30 minutes. A standard calibrated inoculating loop was used to transfer the urine sample onto a cystine lactose electrolyte-deficient (CLED) agar plate. The plates were aerobically incubated at 37°C for 48 hours. A bacterial growth count of more than 10^5^ colony-forming units per milliliter (cfu/mL) was considered significant. Henceforth, standard CLSI guidelines (21) were applied to classify the growth. Once growth was assured, the Kirby-Bauer susceptibility test protocol (22) was used to investigate the antimicrobial resistance to uropathogens. In this study, a total of 17 antibiotics were tested for resistance. These were Amikacin, Amoxicillin/Clavulanic acid, Azithromycin, Aztreonam, Cefixime, Cefoxitin, Cefpodoxime-Proxetil, Ceftazidime, Ceftriaxone, Cefuroxime, Ciprofloxacin, Gentamicin, Levofloxacin, Linezolid, Meropenem, Nitrofurantoin, and Trimethoprim/Sulfamethoxazole. The reason for choosing these is that these antibiotics are commonly prescribed for urinary tract infections in Bangladesh.

## RESULTS AND DISCUSSION

The results are organized as follows: first, we will present the general demographic characteristics of the patients. This will be followed by an analysis of the uropathogens identified, along with the demographic details of the patients infected with each pathogen. Next, we will discuss the antimicrobial resistance observed, including the demographic characteristics of the patients who exhibited resistance. Finally, the report will conclude with a section on the nature of antimicrobial multidrug resistance.

### General demographic nature

The study was conducted over a period of 11 months, from February to November 2022. A total of 7,465 unique patients of all ages and both genders were included in the sample. The general demographic characteristics of the sample are presented in Table 1. According to the table, out of 7,465 patients seeking treatment at the hospital, 4,698 were females and 2,767 were males, representing approximately 63% female and 37% male patients. Among those treated, 800 females and 323 males—a total of 1,123 patients—were diagnosed with urinary tract infections. This results in an infection rate of 17% for females and 11.7% for males. Of the total number of infected patients, 71.2% were females and 28.8% were males. These findings are consistent with a previous study conducted in Bangladesh in 2022 (20).

**TABLE 1:** General nature of the patients in the study sample

Gender	Total treated patients	Percentage of total	Number of infected patients	Infected to total percentage	Percentage of infected patients
Female	4,698	62.9	800	17.0	71.2
Male	2,767	37.1	323	11.7	28.8
Total	7,465		1,123	28.7	

**Fig 1:**
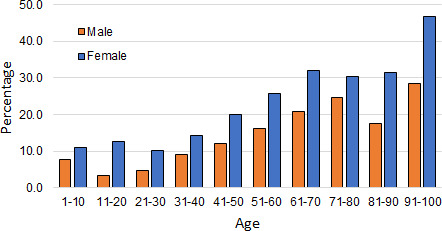
Gender and age distribution of the infected patients as a percentage of treated patients in the study.

**Fig 2:**
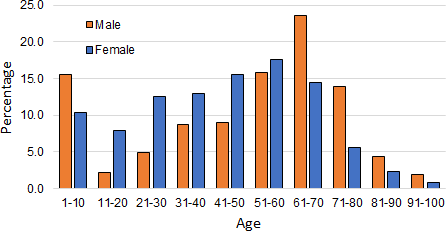
Gender and age distribution of the infected patients as a percentage of total infected patients in the study.

A more detailed demographic nature of the patients is shown in Fig. 1 and 2. Figure 1 reveals that of the treated patients, for all age groups, more female patients are diagnosed with UTI than men. The percentage of infection consistently rises with age for both genders.

Figure 2 shows the age and gender distribution of the patients who have been diagnosed with UTI. The figure shows that prior to the age of 60 years, the incidence of UTI is significantly and consistently higher among females. Several reasons are responsible for this. Among these, the primary reasons are a shorter urethra of women, unhygienic sexual activity, menopause, and lack of acidic fluid (9, 23). After the age of 60 years, males are more vulnerable to UTIs. The primary reason for this is the enlargement of the prostate and unhygienic urinary discharge (24, 25).

Inadvertently, Fig. 2 also reveals that a substantial number of infected patients are infants (aged 1–10 years). Further results from this study have also revealed that a considerable number of patients are in this age group. It was felt that this age group is substantially important, and this group needs to be investigated in considerable detail. Therefore, in the present study, even though some patients will appear in the age group of 1–10 years, we will refrain from making any comment about this age group.

### Uropathogens detected

**Fig 3:**
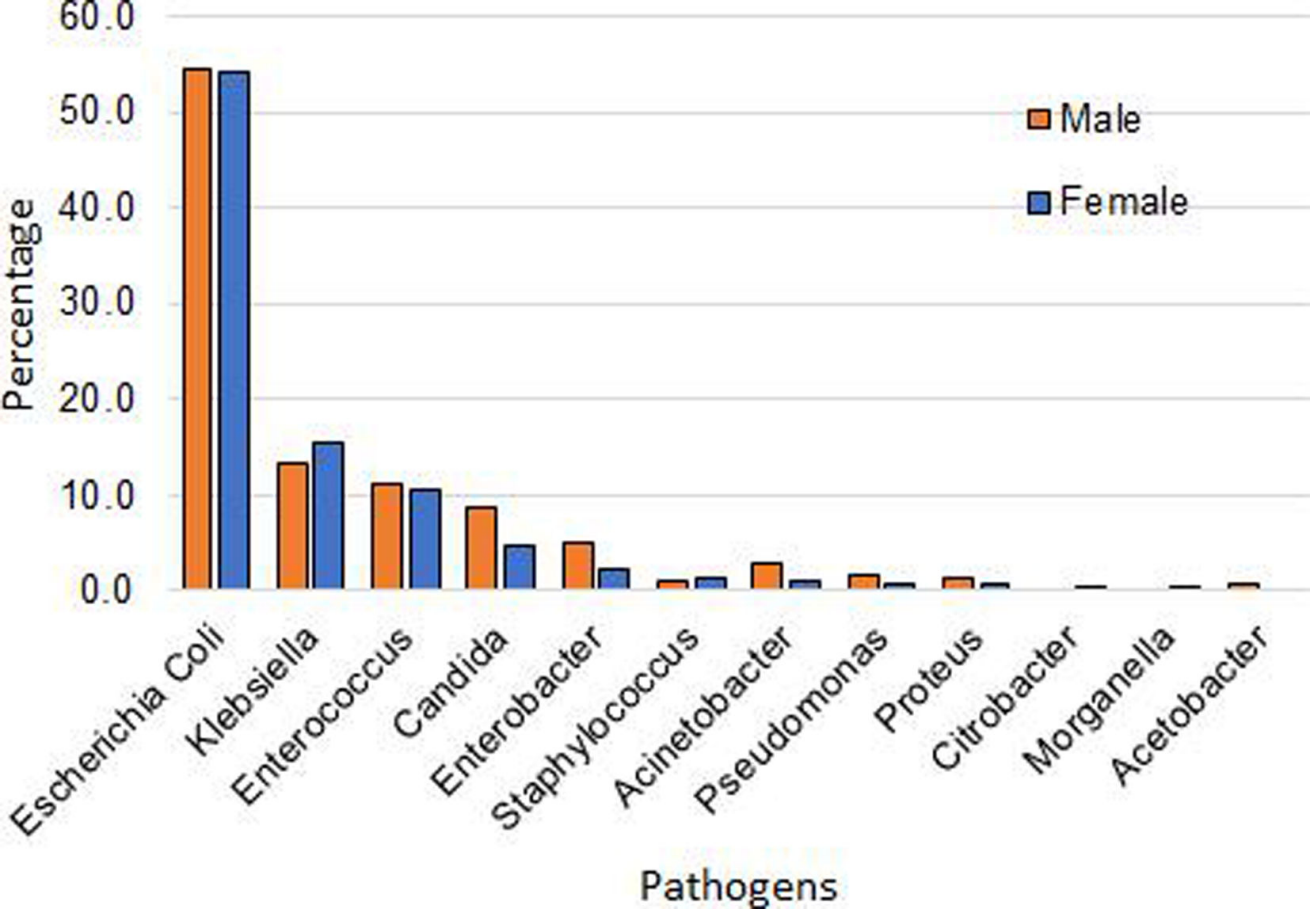
Uropathogens detected and their gender distribution.

The detected uropathogens and their gender distribution are shown in Fig. 3. The top three uropathogens are *E. coli*, *Klebsiella,* and *Enterococcus*. It is quite remarkable that the level of infection of all these uropathogens is of similar levels for both males and females. Notwithstanding, *E. coli* has been found to have the highest level of infection. Approximately 55% of both males and females have been found to be infected with this uropathogen. The next two uropathogens are *Klebsiella* and *Enterococcus*, respectively. Their levels of infections for both genders are approximately 15% and 10%, respectively. These results qualitatively agree with the previous studies conducted in Bangladesh and other countries shown in Table 2. Remarkably, the top two leading uropathogens are the same in all studies except in the study conducted in Bangladesh in 2015. This may be an indication that the nature of the infection may have modified over the years.

**TABLE 2:** Uropathogens detected in some previous studies

Country	Year (reference)	Leading uropathogens and their percentage of detection
Bangladesh	2015 (26)	*Escherichia coli*, 50%; *Staphylococcus*, 16%; *Klebsiella*, 12%; *Enterococcus*, 6%
Bangladesh	2021 (20)	*Escherichia coli*, 62%; *Klebsiella*, 11%; *Staphylococcus*, 6.8%; *Enterococcus*, 6.8%
Spain	2021 (27)	*Escherichia coli*, 56%; *Klebsiella*, 13%; *Enterococcus*, 6.8%; *Pseudomonas*, 7.2%
China	2022 (14)	*Escherichia coli*, 47%; *Klebsiella*, 18%; *Enterococcus*, 16%

**Fig 4:**
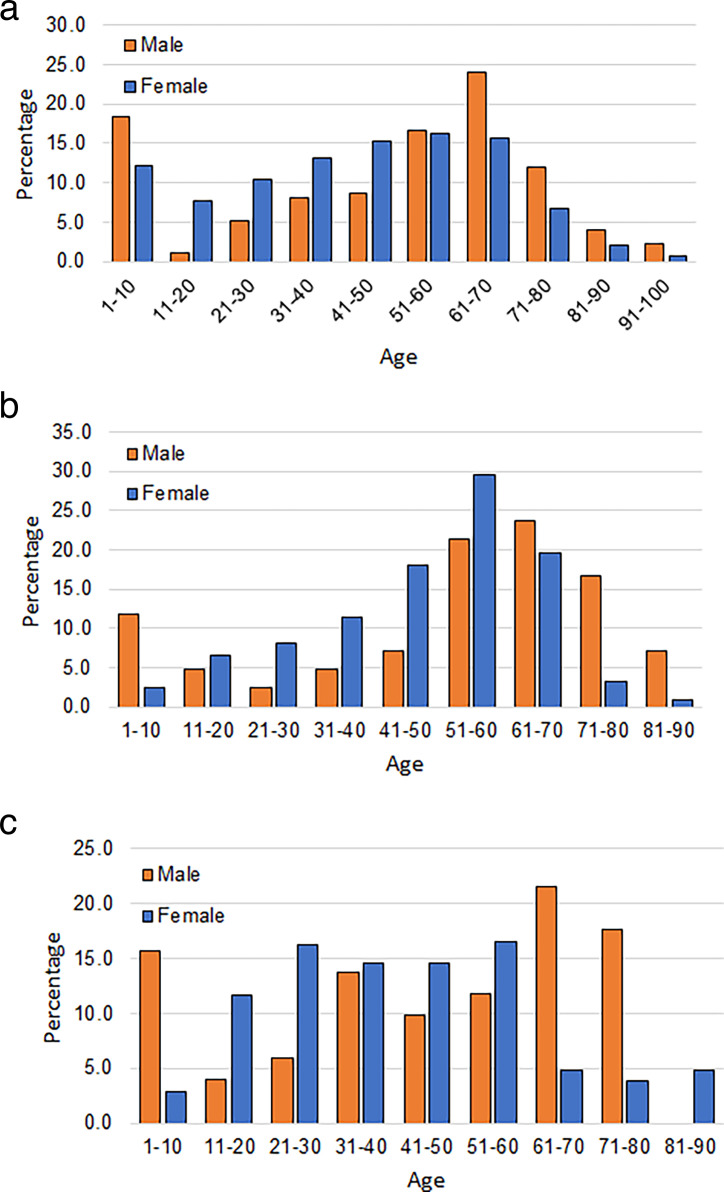
(a) Age distribution of the patients infected with *Escherichia coli* pathogen. **(b)** Age distribution of the patients infected with *Klebsiella* pathogen. **(c)** Age distribution of the patients infected with *Enterococcus* pathogen.

It is expected that the demographic nature of the patients infected with different uropathogens will be different. To understand this demographic nature, the age and gender distribution of the patients infected with the three leading uropathogens is presented in Fig. 4a through c. Even though the qualitative nature of Fig. 4a through c is similar, there are remarkable differences between the three figures. For all three uropathogens, females are more infected before the age of 50 years, while males are more vulnerable after that. But the transition from female to male is qualitatively different for the three uropathogens. Figure 4a shows that, for *E. coli*, the infection nature of females gradually increases with age, reaches a high around the age of 40 years, and remains so until the age of 70 years. This is generally the age of female menopause. Beyond the age of 70 years, the infection rate of females gradually reduces.

The nature of the transition for males is quite different. It is observed that the infection rate of males gradually rises at early age—*albeit* the infection rate remains lower than that of females. But in the age group 50–70 years, the male infection suddenly jumps. We observe that this is generally the age when the male prostate generally begins to grow. We can see a possible reflection of that in the male infection rate. Beyond the age of 70 years, the male infection rate also gradually reduces, though remaining higher than that of females.

Figure 4b shows the results for *Klebsiella* uropathogen. Once again, the figure shows that at a younger age, females are more susceptible to the pathogen. But the pattern of rise with age for *Klebsiella* is more rapid and dramatic than *E. coli*. Especially, in the age group 41–50 years to 51–60 years, the rise is substantially more remarkable than that of *E. coli*. Beyond the age of 60 years, the drop in uropathogen infection is also more rapid.

The *Klebsiella* infection for male populations also follows a similar pattern, but the drop in infection happens generally after the age of 70 years. In general, we observe from Fig. 4b that males are more vulnerable in the age group 50–80 years, whereas females are more vulnerable in the age group 40–70 years.

The gender and age behavior for the pathogen *Enterococcus* is shown in Fig. 4c. We do not see much pattern in this figure, possibly because the number of patients with this infection was not very high. Therefore, we refrain from making any analysis of this figure.

### Antimicrobial resistance

**Fig 5:**
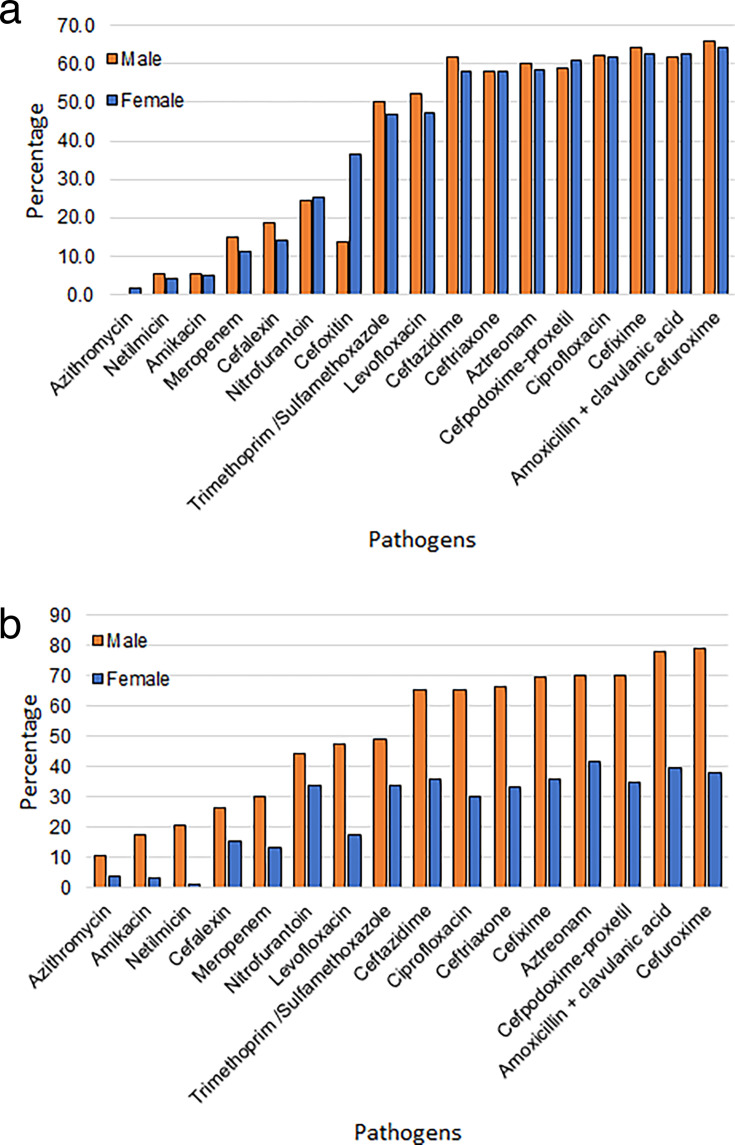
(a) Antimicrobial resistance to *Escherichia coli* to Ciprofloxacin antibiotic. (b) Antimicrobial resistance to *Klebsiella* to Ciprofloxacin antibiotic.

As observed in Fig. 3, several uropathogens were detected in the samples of the infected patients in this study. Here, we present the antimicrobial resistance patterns identified for two dominant uropathogens—*E. coli* and *Klebsiella*. The percentage of patients exhibiting resistance to these pathogens is depicted in Fig. 5a and b.

Figure 5a shows the antimicrobial resistance of *E. coli* to different antibiotics and its gender nature. It was observed that among the patients infected with *E. coli*, least resistance was found to Azithromycin, Netilmicin, and Amikacin—in the range of 5%, while moderate numbers were resistant to Meropenem, Cefalexin, Nitrofurantoin, and Cefoxitin—in the range of 12%–30%. Following this, a substantially large percentage of patients—in the range of 50%–60%—were found resistant to a large number of antibiotics.

The gender nature of *E. coli* resistance shows that for almost all antibiotics, males and females have similar nature of resistance, except Cefoxitin for which females have been found to have remarkably higher resistance than males.

Figure 5b shows the antimicrobial resistance nature of *Klebsiella*. The resistances have been organized based on the percentage of male patients discovered to have resistance to the antibiotic. It is noticed that both males and females infected with *Klebsiella* are least resistant to Azithromycin. The resistance continues to rise for different antibiotics and reaches a level of 60%–70% for more than half of the antibiotics considered in the study. The resistances for females also rise but do not go beyond a level of approximately 35%. This clearly indicates that, comparatively, females have been found to be more responsive to antibiotics against *Klebsiella* for a wide range of antibiotics.

**Fig 6:**
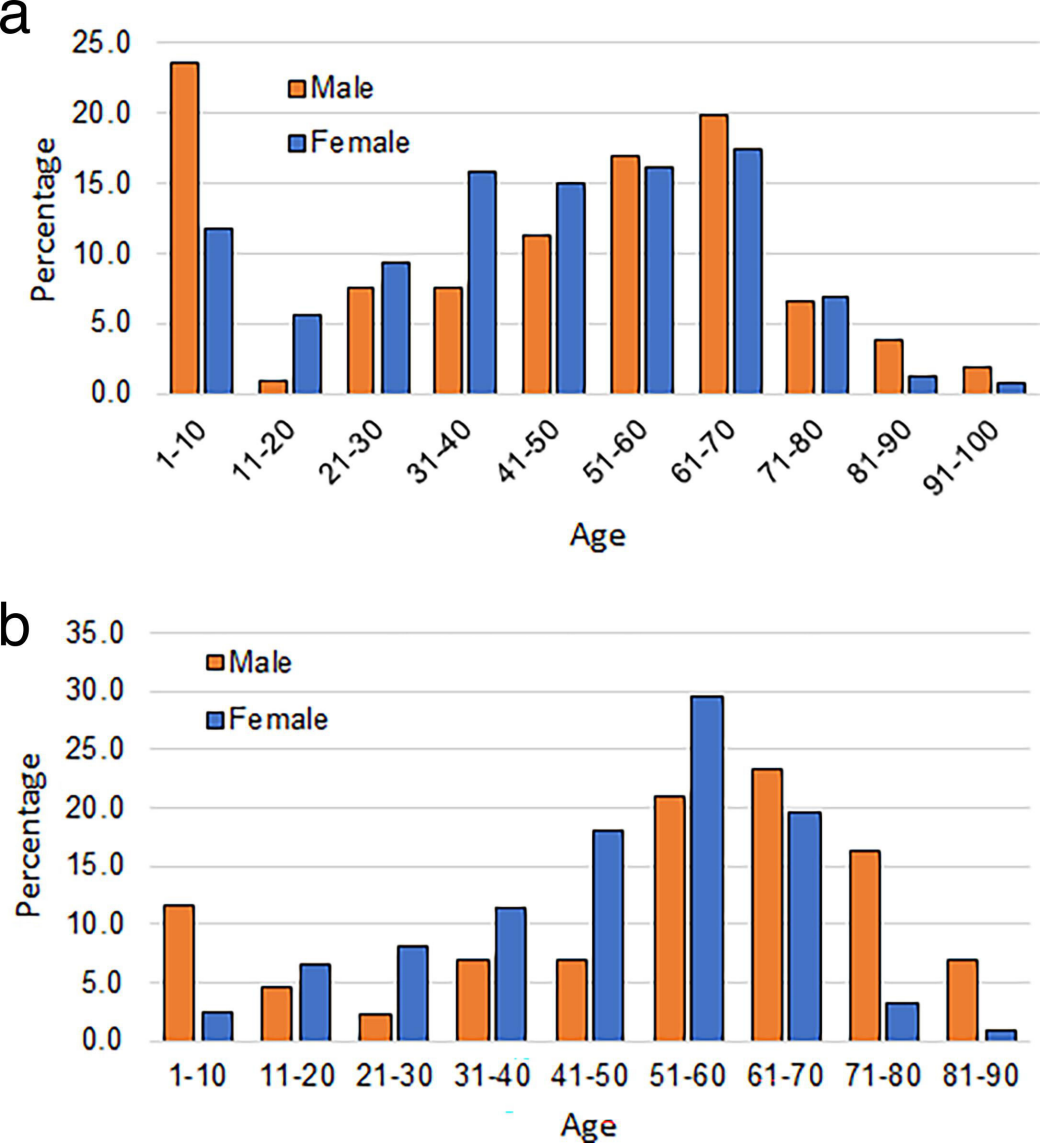
(a) Age distribution of the patients infected with pathogen *Escherichia coli* having resistance to Ciprofloxacin antibiotic. (b) Age distribution of the patients infected with pathogen *Klebsiella* having resistance to Ciprofloxacin antibiotic.

To investigate the age nature of antimicrobial resistance, we had to be judicious. For each antibiotic, the group of patients who are resistant is different. We must start with the premise that their age distribution would be different. Notwithstanding, as we investigated the age distribution of the individual antibiotics, we noticed that their natures are all similar to each other—both for males and females. Because there are a large number of antibiotics, we are not showing them separately; rather, we are showing the age nature of the patients who are resistant to Ciprofloxacin—both for *E. coli* and *Klebsiella*. These are shown in Fig. 6a for *E. coli* and in Fig. 6b for *Klebsiella*.

Figure 6a shows that for *E. coli*, the resistance to Ciprofloxacin for males continues to rise until the age of 70 years, beyond which there is a sharp drop. The age nature of females is quite different. The resistance to the antibiotic grows rapidly with age and remains steady in the age group 30–70 years, beyond which it also drops.

The age distribution of patients infected with *Klebsiella*, who are resistant to Ciprofloxacin, is shown in Fig. 6b. The figure shows that, for males, the resistance to Ciprofloxacin for the pathogen *Klebsiella* remains around 6% until the age of 50 years, beyond which it rises dramatically, and again reduces gradually after the age of 70 years. For females, the resistance of *Klebsiella* to Ciprofloxacin rises gradually with age until the age of 60 years and drops gradually after that.

Previous studies in Bangladesh have not reported the age distribution in this detail. Nevertheless, there is partial qualitative agreement with these results and some results reported earlier (20, 26). However, there are some major differences, especially in the levels of antimicrobial resistance. This only points to the fact that these studies must be done periodically, as they seem to be evolving continuously over the years.

Needless to say, these results are extremely important in the clinical management of cases of UTI.

### Multidrug resistance

The challenge of managing multidrug-resistant pathogens is a continuing challenge (28, 29). Among all the challenges, the challenge of multidrug-resistant urinary tract infection has also been an issue (10, 27, 30). Multidrug resistance in Bangladesh has also received some limited attention (19). We investigated the nature of multidrug resistance of UTI among the patients of this study.

**Fig 7:**
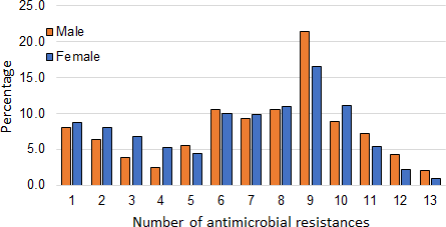
Number of simultaneous antimicrobial resistance.

As stated in the Materials ad Methods, the urine samples were tested for 17 different antibiotics. A substantial number of cases were found that were resistant to more than one antibiotic. The numbers of these multidrug resistances were compiled, and the results are shown in Fig. 7. The figure shows the actual number and percentage of patients with a specific number of simultaneous antibiotic resistances. They also show the gender nature of these number of resistances. For example, the figure shows that the number of patients with resistance to one antibiotic is 8.0% of males and 8.7% of females. The figure shows that approximately 10% of the patients have six, seven, or eight simultaneous antibiotic resistances. There is a sudden unexpected jump in nine simultaneous antibiotic resistances, with the male percentage reaching as high as 20%. It would have been worthwhile to further investigate the nine antibiotics involved in these resistances. Unfortunately, there were only 51 males and 86 females in this group, which would not have given a sensible distribution. What is alarming from Fig. 7 is that within the limitations of the sample size of this study, there were five males and five females who had as high as 13 simultaneous antibiotic resistances.

### Conclusions

The study was conducted to obtain a better understanding of the age and gender nature of antimicrobial resistance to the uropathogens related to urinary tract infections in Bangladesh. The study further confirms that UTI is a continually evolving problem. This study reveals that females before the age of 50 years are more vulnerable to UTI, whereas males are more vulnerable after the age of 70 years. Similar to most previous studies, the two top uropathogens detected were *E. coli* and *Klebsiella*. The differences appear in further uropathogens. This strengthens the argument that studies on antimicrobial resistance to pathogens of UTI are a continuing process. For most commonly administered antibiotics, 50%–60% of the patients were seen to be resistant. The scenario of multidrug resistance was alarming. Almost 20% of the infected patients were found to be resistant to nine antibiotics. A few patients were found to be resistant to as many as 13 antibiotics.
